# Towards a New Paradigm of Non-Captive Research on Cetacean Cognition

**DOI:** 10.1371/journal.pone.0024121

**Published:** 2011-09-07

**Authors:** Lori Marino, Toni Frohoff

**Affiliations:** 1 Department of Psychology, Emory University, Atlanta, Georgia, United States of America; 2 Emory Center for Ethics, Emory University, Atlanta, Georgia, United States of America; 3 TerraMar Research and Learning Institute, Santa Barbara, California, United States of America; University of Sussex, United Kingdom

## Abstract

Contemporary knowledge of impressive neurophysiology and behavior in cetaceans, combined with increasing opportunities for studying free-ranging cetaceans who initiate sociable interaction with humans, are converging to highlight serious ethical considerations and emerging opportunities for a new era of progressive and less-invasive cetacean research. Most research on cetacean cognition has taken place in controlled captive settings, e.g., research labs, marine parks. While these environments afford a certain amount of experimental rigor and logistical control they are fraught with limitations in external validity, impose tremendous stress on the part of the captive animals, and place burdens on populations from which they are often captured. Alternatively, over the past three decades, some researchers have sought to focus their attention on the presence of free-ranging cetacean individuals and groups who have initiated, or chosen to participate in, sociable interactions with humans in the wild. This new approach, defined as Interspecies Collaborative Research between cetacean and human, involves developing novel ways to address research questions under natural conditions and respecting the individual cetacean's autonomy. It also offers a range of potential direct benefits to the cetaceans studied, as well as allowing for unprecedented cognitive and psychological research on sociable mysticetes. Yet stringent precautions are warranted so as to not increase their vulnerability to human activities or pathogens. When conducted in its best and most responsible form, collaborative research with free-ranging cetaceans can deliver methodological innovation and invaluable new insights while not necessitating the ethical and scientific compromises that characterize research in captivity. Further, it is representative of a new epoch in science in which research is designed so that the participating cetaceans are the direct recipients of the benefits.

## Introduction

Cetaceans (dolphins, porpoises and whales) are an order of fully-aquatic mammals who have engrossed scientists and the public alike with their large complex brains, impressive intelligence, and social and communicative sophistication [1]–[3]. For a long time the study of these characteristics in cetaceans lagged behind the rich literature on cognitive, social and even cultural aspects of nonhuman primates. And, just as we have learned that some nonhuman primates possess such qualities as self-awareness, morality, culture, empathy and politics, we now have evidence for similar sophisticated abilities in cetaceans and other animals such as elephants. These developments have provided new lenses through which we have reconsidered these aspects of ourselves; the reference point by which we can view our own characteristics relative to other animals has expanded and diversified beyond the primates. Therefore, the complex sentience of other animals such as cetaceans must be recognized and their physical, psychological and behavioral needs appropriately protected. Accordingly, scientists are now faced with the task of accommodating this contemporary knowledge of cetacean neuroanatomy and behavior in ways that alter their research approaches and priorities.

### Cetacean cognition

Cognition refers to the thought processes of an individual; it typically comprises memory, problem-solving, concept formation, self-awareness, and other abilities that involve information processing at various levels and in various domains. It is important to define the term cognition in the context of our arguments in order to be clear about what kinds of studies we are proposing in this paper. Cognition can be assessed through indirect measures and inference as well as through direct tests. Ongoing long-term field studies of social complexity, foraging, and culture in dolphins and whales continue to yield some of the most intriguing insights into cetacean behavior. Examples include long-term observations of sponge-carrying in bottlenose dolphins (*Tursiops* sp.) in Shark Bay, Western Australia, which have led to the discovery of tool use in dolphins and provided important information about learning and cultural transmission [4]–[6] and work on communication among dolphins and whales which has produced insights into vocal learning and referential signaling in cetaceans in the wild [7]–[9]. Generally, field studies reveal the importance of cetaceans learning and remembering individuals within their community and recognizing their particular characteristics and interrelationships, all abilities reliant upon strong long-term memory and of relevance to cognition [10]. Likewise, neuroanatomical postmortem studies of brain size, structure and complexity in dolphins and whales provide critical information about the neurobiological bases of intelligence and cognition and allow for inferences about these processes that may be tested through behavioral studies [11], [12].

Field and neuroanatomical studies like the ones mentioned above are potentially important sources of relevant data about cognition and promote the generation of hypotheses. But they often do not allow measures of cognitive abilities. In this paper we propose developing ways to more directly assess cognition in wild individuals that may replace studies in captivity and form the basis for a more extensive cognitive ethological approach in cetaceans; one that also encompasses aspects of their behavioral ecology. There are a number of protocols available for studying cognition that either can be potentially transferred to research on dolphins in the wild or are already being applied to study wild individuals, including cetaceans. We describe several below. One of the keys to being able to transfer cognitive tasks from the captive situation to the wild is the opportunity to work with individual dolphins one-on-one. Individuals known as lone sociable dolphins present the potential for doing so. Lone sociable dolphins are free-ranging cetacean individuals who are often solitary, yet, for one reason or another, have initiated, or participated in, sociable interactions with humans in the wild with some regularity [13], [14]. Some of these individuals were orphaned and have become separated from their social group and are truly isolated from conspecifics. Others move back and forth between interactions with humans and members of their own (or other) species but nevertheless fall under the category of lone sociables. There are numerous known individual cetaceans who fit this description; mostly bottlenose dolphins in various regions (*Tursiops truncatus*), beluga whales (*Delphinapterus leucas*) in Eastern Canada and orcas (*Orcinus orca*) in the U.S. Pacific Northwest. And while not all of them will be good candidates for research, many of them can be with the right circumstances and proclivities of the individual dolphin or dolphin group.

### Cognitive Tasks with Cetaceans

Many cognitive tests assess processes such as learning, memory, communication, attention, the ability to discriminate stimuli, imitation, and preferences through basic procedures that involve repeated interactions with particular individuals. If these protocols are developed they could be used to assess cognitive capacities in lone sociable dolphins in the wild. As described later in this paper, interactions with lone sociables may, under certain circumstances, afford the opportunity to present stimuli of various kinds (including mirrors for testing self-awareness), present “choice paradigms” with objects to assess preferences, and initiate simple training procedures that can be used to probe learning and memory and other cognitive abilities. Most of these kinds of tests do not require an extensive experimental set-up but are dependent upon regular access to an individual in a way that allows a certain degree of methodological consistency.

Communication and language research has been one of the most vigorous areas of cetacean study. Studies of natural communication have been ongoing for decades and can be complemented by further work using new technologies and methods. These studies typically involve acoustic and visual recording in order to extract correlations among sounds, behavior and context. Playback experiments - a set of techniques by which natural or synthetic signals are broadcast to an animal or groups of animals and the response noted – are subsequently used to reveal what listeners know about the broadcast signal or the signaller that produced it. These kinds of studies have typically been conducted with groups of cetaceans but can also be applied to individuals in order to learn more about how individuals process communicative sounds. Capabilities to perform this sort of research will grow with our ability to create increasingly sophisticated pattern detection algorithms, present relevant stimuli in playback experiments, and monitor the detailed behavioral responses of subjects underwater.

One of the main goals of research with captive cetaceans has been to determine whether dolphins and other cetaceans can comprehend an artificial symbolic language. The work in captivity heretofore has provided important insights into cetacean intelligence and cognition [15], [16]. However, similar methods can potentially be used to engage individual lone sociable dolphins in tests of language comprehension. For instance, interactive underwater keyboards containing visual symbols that dolphins could select have been used to study these skills in captive dolphins [16], providing a closer approximation to two-way communication. Denise Herzing and her colleagues piloted the use of an underwater keyboard with a habituated group of wild spotted dolphins with some success. Moreover, Herzing and collaborators from Georgia Tech in Atlanta are currently developing a cutting-edge technology that will potentially provide a much more sophisticated interactive interface for human-dolphin communication in the wild. Although these efforts are challenging and not a guarantee of success, they represent the promise of applying new technologies to the study of communication and language comprehension in wild dolphins.

## Analysis

### Why do we need a new paradigm for cetacean cognition research?

There are advantages and disadvantages to studying dolphins and whales in captivity versus the natural setting. Research in captivity affords a level of experimental control and internal validity that cannot be as easily achieved in the natural setting. Decades of research on captive dolphins has resulted in a rich literature on their intelligence, self-awareness, and cognitive abilities [11]. On the other hand, captive studies are limited in external validity for a variety of reasons. These comprise the unknown and largely uncontrollable developmental-cognitive effects of living in an artificial physical, perceptual and social environment on the generalizability of findings to wild cetaceans. Captive studies may be confounded by the physical and psychological stress and trauma evidenced in illnesses and aberrant dolphin behavior described below. Also studies of wild dolphins may reveal behaviors and capacities that are absent or diminished in captivity. Recently, a study of a group of wild chimpanzees revealed that their gestural repertoire was over twice the size suggested by studies of captive chimpanzees [17]. Such studies suggest that captivity may truncate capacities under some circumstances and lead to inaccurate conclusions. On the other hand, there are also difficulties and limitations associated with interacting with wild dolphins and, in particular, lone sociable dolphins, who may not be representative of other dolphins who live in normal wild social groups.

As important as the above pro and con arguments are, there is an arguably more compelling reason to consider adopting a new paradigm for studying cetacean cognition. This has to do with the essential importance of adjusting our behaviors, protocols, and paradigms to the very information provided by our scientific endeavors. In our view, the conclusion from decades of cumulative results of both captive and field studies is that cetaceans possess a level of intelligence, awareness and psychological and emotional sensitivity that makes it unacceptable to continue to keep them in captivity if not necessary for their welfare, survival, or conservation. We do not deny that captive studies have contributed substantially to this conclusion. Our point is that now that we have this knowledge about cetaceans it is incumbent upon us to revise our approaches to studying them.

### How and why captivity harms cetaceans

Captivity for both wild-caught and captive-born cetaceans is devastating on a number of levels ranging from harm to the captive individuals to negative impacts on entire populations in the wild, even when even a small number of individuals are removed from their social groups [18], [19]. There is a copious scientific literature confirming the damaging effects of captivity on dolphin and whale physical health and psychological well-being. The challenges to cetaceans in captivity are numerous beginning with the physical constraints of the artificial enclosures (regardless of how natural they may appear to humans aesthetically) that limit physical exercise and are often harmful in other ways to the cetaceans' distinctive physiology [20]. Confinement impacts social relationships, degrades autonomy through the imposition of an enforced schedule of activity and behavior, causes boredom produced by a relatively sterile and unchanging environment, induces frustration, and inhibits incentives and abilities to carry out natural behaviors such as hunting and traveling. While awareness of how husbandry in cetaceans in captivity can be significantly improved is increasing [21], the abundant evidence for stress, disease and increased mortality in captive cetaceans is an inevitable outcome of such confinement, loss of control and deprivation where dolphins are held subordinate to humans in unnatural physical and social conditions.

#### Aberrant behavior

There is ample anatomical and behavioral evidence that dolphins are not only self-aware but also emotionally sensitive and psychologically complex [2], [3], [22]–[24]. Many captive cetaceans display physiological and behavioral abnormalities indicative of psychological distress and emotional disturbance. These include stereotyped behavior [25]–[27], unresponsiveness, excessive submissiveness, hyper-sexual behavior (towards humans or other dolphins), self-inflicted physical trauma and mutilation [28], stress-induced vomiting [29], compromised immunology [25], [29] and excessive aggressiveness towards other dolphins and humans [22], [30]. One of the more dramatic forms of aberrant behavior in captive cetaceans is evidenced in the long record of orcas and other dolphins killing and seriously injuring humans, other whales, and themselves in captivity [31]–[36]. These statistics are striking considering that there is not a single recorded instance of an orca seriously harming, let alone killing, a human being in the wild. Moreover, serious aggression among orcas in the wild is relatively low and most injuries, e.g., rake marks, are superficial [37]. These discrepancies in aggression and aberrant behavior between cetaceans in the wild and captivity provide particularly clear evidence for psychological and behavioral disturbances in captive orcas.

#### Stress and Disease

Stress derives from many aspects of captivity, not the least of which is that associated with the many changes in social groupings and isolation that occur in captivity. Social relationships play a critical role in the lives and well-being of dolphins and whales. Bottlenose dolphins, orcas, and other cetaceans are not merely gregarious. They form complex societies with dynamic social roles in intricate social networks [18], [38] many with cultural traditions [39], [40]. In the wild individuals can have very strong and long-lasting relationships [41]. In the “resident” orca groups of the Northeast Pacific, both sons and daughters remain with their mothers in their matrilineal cultures [42]. Conflict in the wild is resolved effectively through various means that include dispersion and shifting alliances within large groups of animals [43], an opportunity not afforded by captivity. Social group composition is dynamic and fluid with individuals exerting choice about their associations. In the confines of captivity where social groups are often artificially constructed and transferred in and out of different pools and facilities without choice, and there is not enough room or social support to resolve conflict, dolphins and whales suffer extreme stress that has led to reduced life expectancy [44]. Waples and Gales (2002) state that a decline in fitness, reproductive and physiological problems or even death can be the result of an animal being subjected to stress. There are several cases where stress, social stress in particular, was the probable cause of illness and death in captive bottlenose dolphins [44]. Several studies [44], [45] provide overviews of behavioral measures of dolphin welfare related to stress in captivity.

Furthermore, the U.S. Marine Mammal Inventory Report [46] lists numerous stress-related disorders, such as ulcerative gastritis, perforating ulcer, cardiogenic shock and psychogenic shock as ‘cause of death’ in captive cetaceans, strongly indicating that stress is an important component of captive display. Moreover, recent work shows that handling and transportation of captive dolphins is so stressful that it can decrease their immune system function [47].

#### Mortality

The effects of increased stress and disease in captive cetaceans are evident in the mortality records as well. Up until a few years ago mortality rates were significantly higher in captivity than in known wild populations of bottlenose dolphins. Only recently have survivorship statistics in captivity (6.4%) reached a level not statistically significantly different from that thought to exist in the wild (3.9%) [48]–[53]. The best estimate of average and maximum lifespan for captive and wild bottlenose dolphins is about 25 and 45 years, respectively [51]. But there are biases in these data that make it doubtful that bottlenose dolphins live as long in captivity as in the wild (see below).

Importantly, bottlenose dolphins face a six-fold increase in risk of mortality immediately after capture from the wild and immediately after every transfer between facilities [51]. These findings demonstrate that the stresses associated with transfer from one captive facility to another and capture from the wild are similar.

For orcas the discrepancy in mortality rates between captivity and the wild is even greater. The natural average lifespan for male and female orcas is 29.2 and 50.2 years, respectively, with a maximum longevity of 60 and 90 years, respectively [50], [52]–[55]. In captivity most orcas do not survive much past the age of 20 years ([36] for a review). DeMaster and Drevenak [45] estimated the annual mortality rate for captive orcas at 7.0%, and two further studies, Small and DeMaster (1995) and Woodley et al (1994) both estimated (captive) annual mortality rates at 6.2% (excluding calves) [51], [53], considerably higher than the 2.3% annual mortality rate figure for wild populations [48]. Moreover, there is evidence suggesting belugas die prematurely in captivity as well [56].

It should be noted, when interpreting any of the above findings, that survivorship statistics from captive facilities often exclude periods of sharply increased mortality – those associated with capture and transfer. According to Small and DeMaster (1995) [51] the first 60 days of captivity should not be taken into account when calculating survival rates for wild-born individuals, since the mortality during this time is so high. Further, remote locations and many non-western or developing countries were not included in these studies; hence it is likely that the worst of these facilities were omitted from these data. These biases can easily lead to artificially inflated survivorship data.

All of these findings provide empirical evidence that captivity is harmful to cetaceans, resulting in abnormal behavior, stress-related disease, and, ultimately, high mortality/short lifespans. This state of affairs is not only unfavorable as a context for scientific work it makes the confinement of cetaceans for research purposes difficult to defend ethically.

Given all of the disadvantages of maintaining cetaceans in captivity for research, how should we move forward if we wish to continue learning about and *from* cetaceans? The answer lies in building upon ongoing research in the natural habitat and using these various efforts to create a new paradigm of research on cetacean cognition.

## Results and Discussion

### A New Paradigm of Interspecies Collaborative Research

An ethically and scientifically progressive research paradigm takes into account current knowledge about the complex psychological and sociological needs and capacities of cetaceans as well as the increasing anthropogenic challenges to their survival worldwide. A new era of cetacean research has been developing ‘beneath the surface’ over the past three decades that exemplifies a more responsive approach to what we now know to be key aspects of both individual cetacean wellbeing and conservation. This new approach is called Interspecies Collaborative Research (ICR) [57], [58]. ICR amounts to optimizing existing natural conditions for the primary benefit of the cetacean rather than imposing artificial ones for the sole benefit of the researcher. (Moreover, this new paradigm does not include research on captive animals unless exceptional circumstances exist that involve rehabilitation and eventual transfer to a sanctuary or release to the wild as well as mutual cooperation in the absence of human withholding of positive stimuli or applying negative reinforcement.).

Possibilities for studying free-ranging cetaceans who initiate close proximity and even sociable interactions with humans have been providing unique scientific opportunities for an era of less-invasive cetacean research. Inherent to the methodology of ICR is respect for and protection of cetacean individuals, groups, societies, and cultures. We now know that the survivorship of individuals is inextricably linked to that of their culture [40], [59] and a population's ability to survive may be particularly dependent upon the cultural role of key individuals in their group - so that the concept of wellbeing must encompass all levels of concern ranging from the individual to the society. ICR offers unique insights and methodologies concordant with new empirical data compelling us to reevaluate what is rigorous and ethical science with respect to the individual as well as the conservation of entire populations and species.

Habituation and interspecies sociability in the wild has certainly been explored in prior years by pioneering scientists such as Jane Goodall with chimpanzees in the 1960's and Cynthia Moss with elephants beginning in the 1970's. But the line between the observer and the observed is especially blurred when cetaceans initiate aspects of close proximity and sociability towards human boaters and swimmers. The choice of some free-ranging dolphins and whales (as individuals and in groups) to initiate or participate in sustained physical proximity and even sociable interactions with humans is somewhat unique among wild animals (especially those not provisioned with food). What we knew of as fables of free-ranging dolphins exhibiting sociable behavior towards humans from times of antiquity [60] are realities encountered by modern day researchers.

Interspecies cetacean-human sociability in the wild involves a continuum of behaviors in which cetaceans approach, or are receptive to human approach, and exhibit close and sustained physical proximity with humans that may include prolonged visual and acoustic contact and exchange, and may even involve tactile sociable contact, acoustic and postural mimicry, and play [57], [61]. Cetacean sociality with humans ranges from the extreme of solitary individuals who are geographically isolated from conspecifics (typically, young orcas or belugas who have been orphaned but are nutritionally weaned) to individuals who interact with conspecifics, e.g., bottlenose dolphins (though some are solitary), mother and calf pairs of gray whales (*Eschrichtius robustus*), humpback whales (*Megaptera novaeanglia*) individually or in groups, dwarf minke whales *(Balaenoptera acutorostrata*) in groups, and whole populations of spotted dolphins (*Stenella frontalis*) in the Bahamas.

Unique opportunities for studying cetaceans with deference to their choice, on their terms, and in their own environments are providing alternatives to more invasive methods of scientific investigation.

Over the past few decades, not only have dolphin- and whale-watching become popularized, but even in-water encounters with dolphins and whales have become commercialized in various areas around the world [61]-[65]. The commercial aspects of observing cetaceans in their natural habitat are certainly preferable to the unsustainable (let alone cruel) aspects of intentionally slaughtering. Yet, it is critical to acknowledge that close contact with any wild animals, including dolphins and whales, can present serious risks for cetaceans (and humans) and implementation of precautions are warranted to protect them [66].

### ICR with Solitary Cetaceans

A unique situation arises when cetacean individuals socialize exclusively with humans or have somehow lost contact with and access to conspecifics. When addressing such lone, sociable cetaceans research protocols can and should be developed to do “double-duty” as protection and enrichment on the one hand and data collection on cetacean psychology on the other [67], [68].

In the only on-site assessment of multiple species of solitary, sociable odontocetes (toothed whales, dolphins and porpoises) over numerous years, Frohoff identified three critical aspects of successful protection of solitary individuals (especially apparent when implemented in stewardship programs with Catherine Kinsman designed to protect orphaned and isolated belugas); (1) early assessment of the unique and varied risks encountered by each individual as well as any distinctive behavioral or physical qualities, (2) carefully designed, but nimble and quickly-implemented protocols tailored for each circumstance to mitigate risks to the cetaceans (and sometimes humans), and (3) early implementation of research (including aspects of communicative, cognitive, psychological and emotional complexity) feeding back directly into the second aspect, risk management and promoting wellbeing [66]-[71].

**Figure 1:**
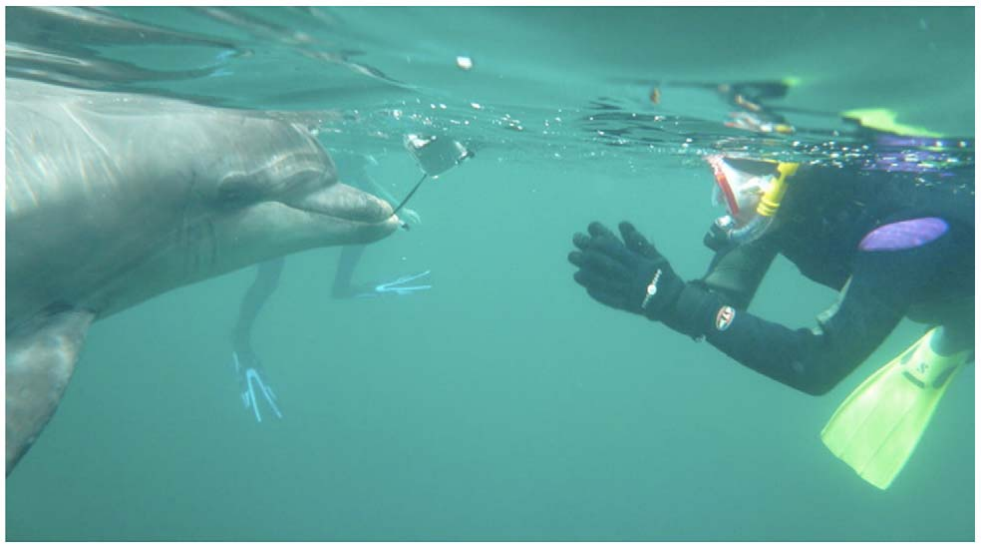
Free-ranging dolphin in the Irish Sea initiating what became a collaborative “choice” study. (Photo: Ute Margreff).

In the past, scientific documentation of sociable interactions with cetaceans has almost entirely been with odontocetes and typically with members of the family Delphinidae, particularly bottlenose dolphins (Figure 1). However, notable exceptions have been documented in the lone, sociable beluga whales (family: Monodontidae) observed annually for the past ten years under the Whale Stewardship Project and TerraMar Research [68], [70] and for two orcas (see various contributors in both [13], [72]). These studies are the first in the world of their kind for orphaned and solitary individuals of these species. In the intensive research efforts for the belugas, it was found that the interactive behaviors of these individuals with humans, boats and other objects were complex and numerous. Frohoff and Kinsman have, to date, collected approximately 500 hours of videotape data for seven individual belugas over a decade. Previously, orphaned and solitary belugas were considered demographic and behavioral anomalies, but their more commonplace occurrences have brought attention to their importance in conservation. With the marked increase in the number of orphans spotted over time, uncertainty about the cause of these orphanings, and a deepened understanding of the role of sociality in overall odontocete population viability, the study and protection of these individuals is of critical conservation concern [67]. The proximal objectives have been to study and support the factors important to each beluga's physical, psychological, and behavioral health and then apply this knowledge to long term conservation.

**Figure 2:**
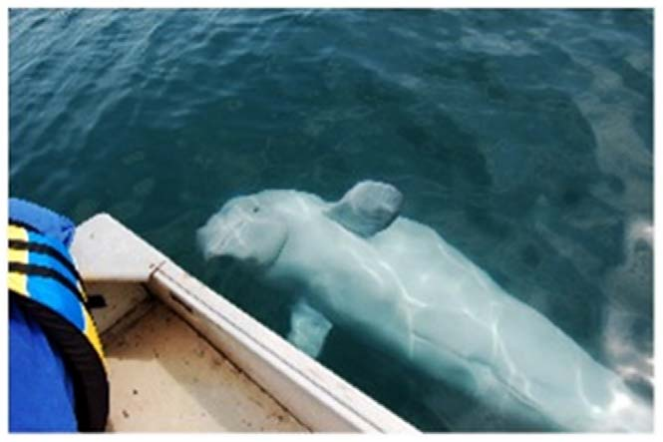
Beluga “Q” observing occupant of boat. (Photo: Catherine Kinsman).

The beluga whale named “Q” is one of several orphaned whales who Frohoff and Kinsman have studied over the past decade. This beluga has not only been monitoring the researchers in turn (as interpreted by frequent approaches and often sustained proximity to us, with prolonged visual and acoustic observations,) but has displayed an astonishing array of interesting behaviors that can be explored to study cognition (Figure 2). For example, Kinsman reported a behavior of interest when reviewing footage from a remotely-operated underwater video camera beneath the boat. She noted: “When you see the beluga looking into the extra-wide lens of the camera, he is apparently watching what is a reflection of himself in that reflective lens.” (C. Kinsman, personal communication 2010). Presentation with a mirror (or playing sounds resembling those made by other cetaceans) to an isolated cetacean individual may mislead them into thinking they are not alone and could potentially therefore be undesirable and unethical. Yet, mirror self-recognition tests may be perfectly reasonable choices for dolphins and whales who are already in the company of conspecifics in the wild.

### ICR Research with Cetacean Groups

In addition to the study of cognition and other aspects of individual psychology, ICR has also been developed through ongoing efforts to study habituated groups of wild cetaceans. One such example is that of The Wild Dolphin Project, led by Denise Herzing. This research, in its 25th year, involves observations and interactions with a habituated group of wild Atlantic spotted dolphins (*Stenella frontalis*) in the Bahamas. These spotted dolphins also frequently swim with bottlenose dolphins affording the opportunity to observe natural inter-species dolphin behavior. The goal is to develop a two-way communication system between humans and dolphins and to accomplish these studies with the least amount of invasiveness possible and, importantly, on the dolphins' own terms. This research uses the psychological model of distributed cognition, using observable and measurable phenomena to infer the flow of information in a group of cetaceans. Anticipating a watershed change in cognitive research on dolphins. Herzing and Johnson (2006, p.554) [73] wrote:

“Data from observational settings may be critical… when the cognitive laboratories of the past no longer exist or no longer conduct experimental cognitive work.”

Another example of ICR that combines protection and research in the wild is that of the Orca Research Trust, lead by Ingrid Visser in New Zealand and other parts of the world where orcas are found. This work provides proof of concept that important research can be done with individual as well as groups of wild orcas. This research project led to recent detailed reports of a special type of cooperative hunting among orcas in which they work together to create waves to displace penguins and seals on ice floes [74]. These findings, along with other similar reports, provide insight into the cognitive capacities of orcas. Much of the research done by Visser involves interaction with habituated individual orcas as well – an approach made possible by engaging the orcas in their familiar natural habitat.

**Figure 3:**
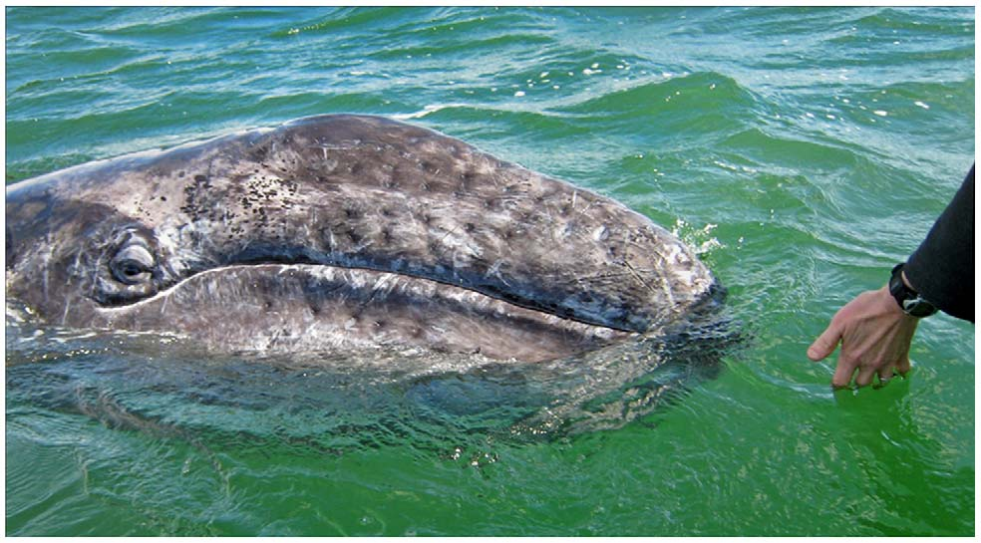
Frohoff studying cognitive and communicative aspects of "friendly" gray whale behavior is an example of the unprecedented research opportunities for collaborative research in mysticetes (baleen whales). Photo: Toni Frohoff.

ICR presents unprecedented opportunities for studying cognition and psychology in mysticetes (baleen whales) as well. In some parts of the world, gray, minke, and humpback whales are well known for their “friendly” behavior towards humans; although the degree and form of attraction and sociability towards humans varies widely across whale individuals, species, and locations. In the Baja lagoons in Mexico where the northwest Eastern Pacific gray whales migrate annually to breed and give birth, a tradition of sorts has developed over the past few decades in which some whales often initiate prolonged visual and even tactile interaction with boaters [75], [76]. After having documented aspects of this unique type of interspecies communication, Frohoff has been exploring the potential for cognitive and psychological studies; including carefully implemented mirror response studies with the whales who approach the small boats; a rare opportunity for looking into the minds of mysticetes in a minimally intrusive way and one that may yield results supporting their increased protection (Figure 3).

The potential for collaborative research between the two species is beautifully illustrated in these lagoons; particularly when regulations honoring the need for space and privacy for the whales are judiciously self-enforced by the boat operators and whale watchers. Also, one of the best examples of the benefits of wildlife-tourism dollars on wildlife conservation can be found here given the notable influence of these funds on the government protection of these lagoons for the whales [76]. The need for reciprocity inherent in research, let alone in whale-watching, is perhaps no more clear than when mother whales bring their calves and initiate and seem to encourage gentle touch and even play with boaters. Also warranting respect are situations in which humpback whales and minke whales in other parts of the world not only tolerate, but sometimes approach humans in the water. Frohoff (in progress) is currently documenting the in-water interactions that are occurring between humans and humpback whales and analyzing them alone and also relative to in-water human interactions with free-ranging groups of odontocetes she has studied (including spotted, spinner, and bottlenose dolphins). Accordingly, while studying the cognitive and communicative aspects of these interactions, the concordant research goal is to assess the differential impacts of contact with humans and to encourage the amelioration of any identified negative effects and expanding on those that may be positive for these “friendly” cetaceans.

### Caveats and Precautions

Despite the range of potential direct benefits to the cetaceans studied, this new research paradigm is not without its need for stringent precautionary measures for their protection. The same risks inherent in direct or indirect recreational interaction with these cetaceans need to be carefully considered and mitigated. For example, care needs to be taken that habituation, or the positive reinforcement of increased habituation, of free-ranging animals does not occur (except in unique circumstances) given the clearly demonstrated dangers that such misplaced trust in humans can have for cetaceans. And exposure to humans can also increase susceptibility to pathogens [77], [78] just as there are health risks for marine mammal workers handling diseased cetaceans [79]. But Geraci and Ridgway (1991) stated that microorganisms introduced into a pre-existing microbial pool – such as would naturally exist in the wild – would have “no particular benefit or harm to a healthy, immunologically competent animal” [77, p. 192]. Common sense suggests that disease transmission risks for dolphins in the wild are much less than in confined quarters due to the dispersal of microorganisms in an open environment. Also, risks of brief tactile interactions would be mitigated by healthy human investigators who would avoid mucus membranes and other inappropriate touching such as is often observed when these lone sociables interact with the general public [13]. Taken together, although there is always a risk of disease transmission and injury, well-controlled interactions between professionals and cetaceans in open waters represents the least risky scenario when compared with those in captivity where micro-organisms are more concentrated and cetaceans are stressed and confined.

In addition, the integrity of the research methods themselves needs to be maintained through creative procedures that will render meaningful data in the wild. Methodological and logistical challenges to the human researcher working in the natural environment can be intensive anytime, but especially so when responding respectfully to the often unexpected choices and timing initiated by the cetaceans. Such spontaneous events (some involving real-time participation) require that researchers be flexible, and highly prepared for the rapid and unexpected changes that occur in the natural setting. Because no aspect of the situation is controlled, copious detailed records must always be maintained in order to preserve the validity and reliability of the observations.

Just as the psychological and emotional wellbeing of solitary and orphaned individuals is likely much more fragile and precarious than that of any other type of free-ranging cetacean (see above mentioned ethical concerns about mirror self recognition and auditory experiments), so is their vulnerability to serious injury and early mortality, especially in the case of young individuals and solitary belugas and orcas (who seem to be particularly susceptible to injuries from boat propellers) [67], [68]. Therefore, implementation of judicious precautionary welfare measures should take priority in these contexts. Yet because of the unique needs of these individuals, they may be in the most need for the potential benefits of enrichment and support that collaborative research may provide for them. Regardless, precautionary measures in any context are always good practice if such research is to yield benefits for the individuals, let alone the species.

### Moving Forward: Integrating Research with Ethical Responsibility

There is no single and comprehensive prescription for the design of protocols for ICR, yet some commonalities have been observed across species and situations that can inform this approach. In groups of cetaceans as well as solitary individuals who interact closely with humans, research has shown that successful efforts are directly related to early implementation and consistency of on-site, pro-active protection and research programs [66], [68]. Studies on lone, sociable cetaceans of various species such as bottlenose dolphins and beluga whales [62], [68], [69] have shown that ongoing, systematic research integrated with responsible decisions about protection and conservation provide vital feedback on how our interactions with cetaceans affect their welfare.

The suitability and feasibility for the release of various dolphins to the wild has been debated also and almost as much as has the conservation and welfare related ethics of capturing free ranging dolphins for captivity. Yet if some dolphins are considered unequivocally to be non-releasable to the wild once captive (for health or survival reasons), the question of what is best for them should be at the forefront of consideration. Sanctuaries exist for virtually every type of animal, both domestic and wild, except for small cetaceans. A true dolphin sanctuary, defined as being created and operated primarily for the benefit of the dolphins rather than for the gain of people, has not yet been formally created. Sanctuary-living would be preferable to the stresses of being maintained in the confinement of artificial tanks. Any knowledge gained about dolphins and whales while in sanctuary is of value and importance but, as with wild individuals, collected on the cetaceans' terms.

### Limited Resources for Critical Needs

Resources have been severely limited for ICR programs. Only about 20% of the videotaped data on solitary, sociable belugas have been quantitatively analyzed to date and the researchers … “have been unable to capitalize on numerous unique opportunities to implement wild-based studies in areas such as cognition and acoustics” (66 pg. 27). Attempts by Frohoff and others to implement protection and enrichment programs coinciding with research, such as was in the case of an orphaned and solitary orca named “Luna” (who was subsequently killed by a boat's propeller), as well as for various belugas and other habituated cetaceans, have been thwarted by outdated policies and limited funding. But with an infusion of support, ICR is poised to open up new avenues of understanding between humans and cetaceans.

When conducted responsibly, ICR is a collaborative endeavor with other species that creates a two-way lens of observation, i.e. it is the humans who are also being observed and the other species are afforded at least as much choice in participating as the researchers. This approach can open up unparalleled opportunities for obtaining data about normative aspects of cetacean behavior, lifestyle, culture, and some of the more subtle and nuanced, yet vital, aspects of cetacean cognition, communication, emotion, sociality, and behavioral ecology. Moreover, this method also allows for cognitive research on the larger sociable mysticetes which has been, up to now, almost nonexistent. When conducted in its best, most rigorous, and most conscientious form, interspecies collaborative research with free-ranging cetaceans can deliver methodological innovation and invaluable new insights without the ethical and scientific compromises that characterize research in captivity. Researchers may be surprised at what we can learn not only from cetaceans and other animals, but also about ourselves as a species, particularly as we relate to the natural world.
